# CAVO Inhibits Airway Inflammation and ILC2s in OVA-Induced Murine Asthma Mice

**DOI:** 10.1155/2023/8783078

**Published:** 2023-01-10

**Authors:** Feng Huang, Xiaoyun Tong, Chunyan Hu, Qiushi Zhang, Yijie Wei, Min Hu, Lingqi Kong, Rongbing Fu, Xiaohong Li, Yuhuan Xie, Xi Ming, Bojun Chen, Yuping Lin, Lei Xiong

**Affiliations:** ^1^School of Chinese Materia Medica & Yunnan Key Laboratory of Southern Medicine Utilization, Yunnan University of Chinese Medicine, Kunming, China; ^2^The First Affiliated Hospital of Yunnan University of Chinese Medicine, Yunnan University of Chinese Medicine, Kunming, China; ^3^Department of Pharmacy, Tengchong Hospital of Chinese Medicine, Baoshan, China; ^4^Department of Ethnic Medicine, Youjiang Medical University for Nationalities, Baise, China; ^5^Basic Medical School, Yunnan University of Chinese Medicine, Kunming, China

## Abstract

Cang-ai volatile oil (CAVO) is an aromatic Chinese medicine and is widely used to treat upper respiratory tract infections in children. However, the mechanism of CAVO in asthma treatment is unclear. In this study, we investigated the effects of CAVO on airway inflammation and the mechanism of inhibiting Group-2 innate lymphoid cells (ILC2s) in asthmatic mice, which was induced with Ovalbumin (OVA). CAVO improved AHR and airway inflammation in asthmatic mice. CAVO reduced the production of interleukin (IL)-2, IL-4, IL-5, IL-6, IL-7, IL-9, IL-13, IL-25, IL-33, and thymic stromal lymphopoietin (TSLP) in the bronchoalveolar lavage fluid (BALF), while increased the production of IL-10, significantly. CAVO also inhibited the suppressor of tumorigenicity 2 (ST2) and IL-33 expressions in the lung tissue. Moreover, flow analyses demonstrated that CAVO inhibited ILC2s activation by reducing the sedimentation of its upstream cytokines, thus alleviating downstream cytokines. This could be because of the downregulated microRNA-155 and upregulated microRNA-146a. CAVO inhibits ILC2s activation, thus further attenuating airway inflammation and AHR in asthmatic mice. These effects may be related to the downregulation of microRNA-155 and upregulation of microRNA-146a.

## 1. Introduction

Asthma is one of the most intractable chronic airway inflammatory diseases. Its symptoms are mainly characterized by repeated wheezing, cough, chest tightness, etc. Asthma is associated with reversible expiratory airflow limitation, chronic airway inflammation, and AHR [1, 2]. Asthma is often caused by genetic factors and environmental stimuli. The incidence and mortality rates of asthma are increasing worldwide due to the aggravation of environmental pollution. This has become a serious threat to public health. Over 300 million people worldwide suffer from asthma, especially in China (over 45 million) [3]. The number of asthma patients globally may increase to 400 million by 2025, according to World Health Organization (WHO) [4].

Asthma is caused by immunological, genetic, environmental, and pathogenic allergens. Various immune cells and cytokines coparticipate in asthma patients, leading to high heterogeneity from pathologic processes to clinical manifestations [5]. T helper 2 cells (Th2 cells) can produce IL-4, IL-5, IL-9, and IL-13 cytokines, which promote airway eosinophilia, bronchial hyperresponsiveness, mucus overproduction, and airway remodeling in asthma patients [6]. Meanwhile, these cytokines are upregulated in BALF, peripheral blood, and bronchial mucosa of asthma patients [7]. Group-2 innate lymphoid cells (ILC2s) are mainly involved in type immune responses by secreting Th2 cytokines to play a key role in allergic asthma [8].

ILC2s, belonging to lymphoid lineage cells, are derived from common lymphoid progenitors [9]. ILC2s have been found in many tissues, such as the liver, spleen, bone marrow, mesenteric lymph nodes, lung, and peritoneum, especially in the lung tissue, involved in the lung inflammatory response [10, 11]. ILC2s are independent of the acquired immune system and can be activated by various stimuli, including IL-25, IL-33, and TSLP which are secreted by epithelial cells [12]. The activation of ILC2s can directly respond to various external stimuli and secrete type 2 cytokines, such as IL-4, IL-5, and IL-13. IL-33 belongs to the IL-1 family, and its expression is positively related to asthma severity. IL-33 also is a key bridge between the acquired immune system and the innate immune system [3]. ILC2s can express ST2, CD127, CD90, CD45, c-Kit, and killer cell lectin-like receptor G1 (KLRG1). ST2 is a component of the IL-33 receptor and can help IL-33 to exert its function, which is commonly used as a marker for ILC2s identification. KLRG1 can induce ILC2s proliferation and IL13 production [13, 14]. Recent asthma studies have indicated that ILC2s initiate and enhance allergic airway inflammation and steroid resistance [15–17]. miR-155 can directly inhibit ILC2s proliferation and regulate ILC2s cytokine secretion function in allergen-induced inflammation. However, miR-146a can regulate ILC2s function by inhibiting downstream ST2 signaling [18, 19].

Since the outbreak of coronavirus disease 2019 (COVID-19), traditional Chinese medicine (TCM) and its ingredients have been widely used in China as a treatment method for COVID-19 symptoms. Meanwhile, there was founded that TCM and its ingredients were effective activity to regulate immunology and the respiratory [20, 21]. Cang-ai volatile oil (CAVO) is a traditional Chinese medicine (TCM) based on the clinical medication experience of professor Xiong Lei. CAVO is extracted from *Atractylodes lancea*, *Ambrosia artemisiifolia*, *Agastache rugosa*, *Eupatorium fortunei*, *Agastache rugosa*, etc. CAVO can regulate T-lymphocyte immune response to antirespiratory agents [22, 23]. CAVO has been mainly to treat lung inflammation through inhalation. However, its mechanism is unknown. CAVO is an aromatic herbal medicine that has been widely used to treat asthma for many years. This study is aimed at investigating whether CAVO can ameliorate asthmatic AHR by inhibiting ILC2s.

## 2. Materials and Methods

### 2.1. CAVO Preparation

The drugs needed for CAVO preparation were obtained from the Yunnan University of Chinese medicine clinic and identified by Prof. Zhang Jie in the School of Chinese Materia Medica of Yunnan University of Chinese Medicine. CAVO was extracted from the *Atractylodes lancea* roots (100 g), *Ambrosia artemisiifolia* leaves (100 g), *Agastache rugosa* leaves (50 g), *Eupatorium fortunei* leaves (50 g), flower bud of *Agastache rugosa* (50 g), *Zanthoxylum bungeanum* fruits (50 g), *Amomum kravanh* fruits (50 g), *Elsholtzia ciliata* leaves (50 g), *Acorus tatarinowii* roots (50 g), and *Kaempferia galanga* roots (50 g) via hydrodistillation. These samples were distilled eight times using water (v/w) for 1 h, then boiled for 2 h. The water was removed using anhydrous sodium sulfate, and golden yellow liquid with an aromatic smell was obtained. The prepared CAVO (yield; 3.6% (v/w)) was kept in the refrigerator at 4°C for further analysis [22].

### 2.2. Animals and Experimental Study Design

Female BALB/c mice (SPF grade, 5-6 weeks old, and body weight; 18-22 g) were obtained from Liaoning Changsheng Biotechnology Co. Ltd. (SCXK (Liao) 2015-0001, Liaoning, China). The mice were kept in the barrier environment of the experimental animal center of Yunnan University of Chinese medicine (SYXK (Dian) k2017-005) (temperature: 22~24°C and humidity: 45~55%). The mice were given water and food freely and allowed to adapt for one week before the experiment started. This study was carried out in accordance with the recommendations of the “Chinese Society of Experimental Animals.” The approval of animal Ethics was approved by the Committee on the Ethics of Animal Experiment of Yunnan University of Chinese Medicine, Kunming, China (No. R-062021155). Thirty healthy female BABL/c mice were randomly divided into five groups: control group, model group, dexamethasone group (DEX), high dose of CAVO group (60 mg/kg), and low dose of CAVO group (30 mg/kg) (six mice in each group). The mice in the control group were injected with 0.2 mL saline, while mice in other groups were sensitized with 0.2 mL OVA (1 mg/ml ovalbumin and 5 mg/mL Al(OH)_3_) on days 1, 8, and 15. Mice in the control group and the model group were orally given saline (10 mL/kg/d) from day 21, while the mice in the DEX, the high-dose group (60 mg/kg), and the low-dose group were orally given dexamethasone solution (1 mg/kg/d), 60 mg/kg/d CAVO, and 30 mg/kg/d CAVO, respectively. After 1 h, the mice were challenged with an aerosol of 2% OVA excitation solution for 30 minutes, but the control group was challenged with saline alone (once a day, for seven consecutive days). After 24 hours of the last OVA treatment, the mice were sacrificed with carbon dioxide (CO_2_) asphyxia. The mice were in a specific close room, which lead to CO_2_ flow (30%~70% of the cage volume/min), and then, the mice were rapidly unconscious and died caused by CO_2_ overdose.

### 2.3. Measurement of AHR

After the last OVA treatment, the mice had anesthetized with injected intraperitoneally pentobarbital sodium (45 mg/kg). When the mice were anesthetized, they were connected to the Resistance and Compliance System (RC system, BUXCO, Wilmington, NC, USA). Airway responsiveness was expressed as the enhanced pause, while basal readings were obtained and averaged for a 3 min period. Subsequently, different concentrations of methacholine (0, 3.125, 6.25, 12.5, 25, and 50 mg/mL) were aerosolized for 3 min. Readings were obtained and averaged for 3 min after each nebulization. The inspiratory resistance (IR) was measured at every dose to calculate the percentage of each concentration to the baseline.

### 2.4. Histology Analysis and Immunohistochemistry

Left lungs were removed from mice in each group, washed with phosphate-buffered saline (PBS), then dried with filter paper. The samples were fixed in 4% paraformaldehyde for 24 h, then embedded in paraffin, and cut into 3 *μ*m thick sections. H&E and PAS stainings (Wuhan Servicebio Technology Co., Ltd., Wuhan, China) were used to observe the thickening/inflammatory infiltration of the lung tissue and the proliferation of goblet cells around the trachea, respectively. The paraffin sections were dewaxed, then incubated in 3% hydrogen peroxide solution. BSA (3%) was then added to the solution for 30 min. The blocking fluid was removed, and the sample was incubated with a primary antibody. The sections were dried, then incubated with a secondary antibody (HRP-labeled) (Wuhan Servicebio Technology Co., Ltd., Wuhan, China). Finally, the sample was examined using an optical microscope.

### 2.5. Measurement of Cytokines in Bronchoalveolar Lavage Fluid

The mice were sacrificed after 24 hours of the last OVA treatment. The chest was exposed for endotracheal intubation. The right lung was clipped with a vascular clamp for BALF collection via endotracheal intubation using a catheter followed by lavage with 0.3 mL of PBS (thrice). The BALF was centrifuged at 800 × *g* and 4°C for 15 min to obtain supernatant. The levels of TSLP, IL-2, IL-7, IL-25, IL-33, IL-4, IL-5, IL-6, IL-9, IL-13, total serum IgE (Multisciences Biotech Co., Ltd., Hangzhou, China), ST2 (Jiangsu Meimian Biotechnology Co., Ltd, Jiangsu, China), and MUC5AC (Elabscience Biotechnology Co., Ltd, Wuhan, China) in the supernatant were detected using the ELISA kit following the kit's instructions.

### 2.6. mRNA Extraction and Quantitative Real-Time PCR

miRNA Isolation Kit (Tiangen Biotech Co., Beijing, China) was used to extract total miRNA from the lungs. cDNA was synthesized using the TaqMan miRNA Reverse Transcription Kit. TaqMan hsa-miR-146a and 155 assays (Thermo Fisher, Waltham, MA USA) were used to quantify miR-146a and 155 levels. Quantitative Real-Time PCR was performed using LightCycler® 480 II Real-time PCR system (Bio-Rad, Hercules, USA). The following primers were used: Mmu-miR-155 (5′-GTCGTATCCAGTGCAGGGTCCGAGGTATTCGCACTGG ATACGACACCCCTA-3′ and 5′-CCTCGTTAATGCTAATTGTGA-3′) and Mmu-miR-146a (5′-GTCGTATCCAGTGCAGGGTCCGAGGTATTCGCACTGGATACGACAACCCA-3′ and 5′-CGCGTGAGAACTGAATTCCATG-3′).

### 2.7. Flow Cytometry for ILC2s

Lung tissues were digested in 50 *μ*g/mL Liberase^TM^ (1 : 100) +1 *μ*g/mL DNase I (1 : 200), and then the cells were collected. Mouse FITC-CD45 antibody (BD Pharmingen, San Diego, CA, USA) was used to stain the surface markers. The cells were fixated and permeabilized using some antibodies (lineage-APC Streptavidin (BD Pharmingen) markers (CD3*ε* (BD Pharmingen), CD4 (eBioscience, San Diego, CA, USA), CD5 (BD Pharmingen), CD8a (BD Pharmingen), CD11c (eBioscience), FceRIa (BioLegend), CD19 (eBioscience), NK1.1 (BioLegend), F4/80 (eBioscience), TER119 (eBioscience), Ly-6G and Ly-6C (Gr-1, BD Pharmingen), anti-7AAD (BD Pharmingen), anti-CD45-APCcy7 (BD Pharmingen), anti-ST2-PE (BD Pharmingen), and anti-CD90.2 (BD Pharmingen) for ILC2s detection.

### 2.8. Statistical Analysis

Data are expressed as mean ± SD of triplicate experiments. GraphPad Prism 8.0 (GraphPad, San Diego, CA, USA) was used to analyze data and draw images. A one-way analysis of variance (ANOVA) followed by Bonferroni was used to determine statistically significant values. Statistical significance was set at *P* < 0.05.

## 3. Results

### 3.1. CAVO Alleviates AHR in Asthmatic Mic

**Figure 1:**
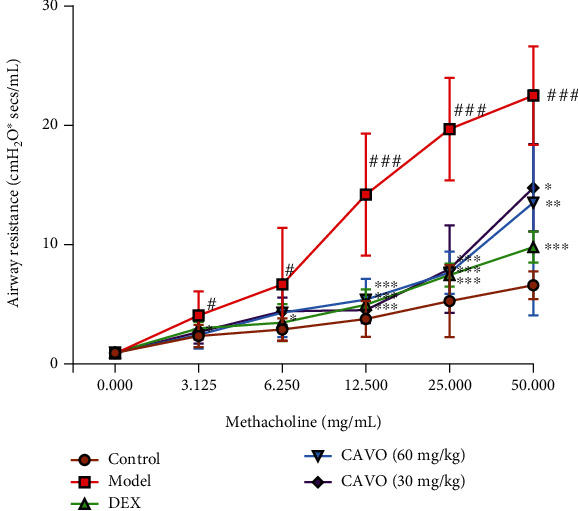
CAVO alleviates airway hyperresponsiveness in asthmatic mice. Data are presented as means ± SEM (*n* = 6, per group, ^###^*P* < 0.001 vs. control group; ^∗∗∗^*P* < 0.001, ^∗∗^*P* < 0.01, and ^∗^*P* < 0.05 vs. model group).

Asthma is mainly characterized by AHR. Herein, a mouse asthma model was induced using nebulized OVA to investigate the effect of CAVO on AHR. The protective effects of CAVO on asthmatic AHR were also investigated using the bronchial provocation test (Figure 1). Airway resistance was significantly higher in the model group than that in the control group, indicating that the asthmatic model was successfully established. However, low and high doses of CAVO significantly reduced airway resistance compared with the model group.

### 3.2. CAVO Attenuates Airway Inflammation in Asthmatic Mice

**Figure 2:**
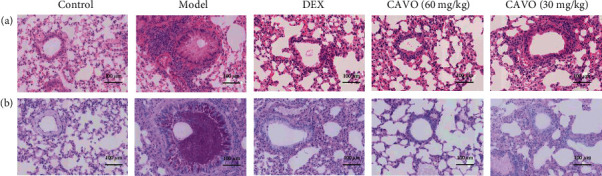
CAVO attenuates airway inflammation and goblet cell proliferation in asthmatic mice (200×, *n* = 6). (a) H&E staining on lung tissues. (b) PAS staining on lung tissues.

H&E staining was performed 14 days after CAVO treatment to evaluate airway inflammation of asthma mice. Many inflammatory cells were infiltrated around the trachea in the lung tissue, while the alveolar wall was thickened and fluid was extravasated (Figure 2(a)). Compared with the model group, dexamethasone and CAVO reduced inflammatory cell infiltration, wall thickening, and epithelial hyperplasia. These results indicate that CAVO can attenuate asthmatic symptoms by deducing airway inflammation in the lungs.

### 3.3. CAVO Alleviates Goblet Cell Proliferation in Asthmatic Mice

Goblet cells and their acidic mucus showed purple-blue particles after PAS staining. The control group had no goblet cells in the airway epithelium, and mucus was not clear in the airway. In contrast, mucus filled the airway, and the epithelium had large goblet cells in the model group (Figure 2(b)). CAVO treatment significantly reduced the number of goblet cells and mucus in the airway. These results suggest that CAVO can reduce the proliferation of goblet cells and mucus production in the airway of asthmatic mice.

### 3.4. CAVO Reduces the Upstream Factors Related to ILC2s in the BALF and Lung Tissue of Asthmatic Mice

**Figure 3:**
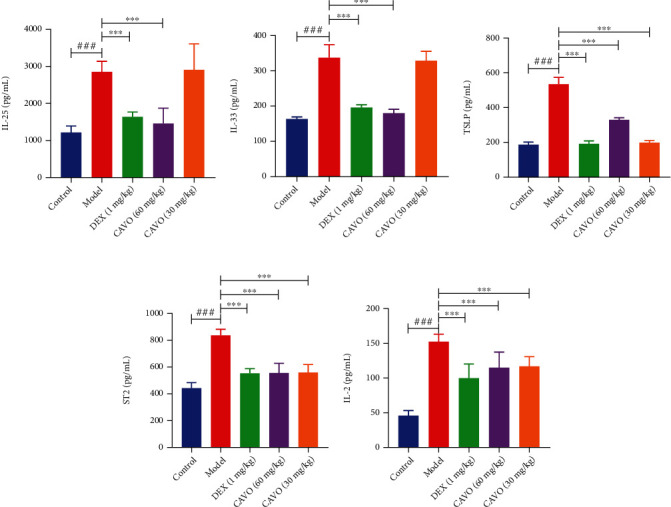
CAVO reduces the upstream factors related to ILC2s in the BALF of asthmatic mice. The levels of cytokines IL-2 (a), IL-7 (b), TSLP (c), IL-25 (d), and IL-33 (e) in the BALF were measured after treatment with CAVO or DEX. Data are expressed as mean ± SEM. (*n* = 6 mice per group, ^###^*P* < 0.001 vs. control group; ^∗∗∗^*P* < 0.001 vs. model group).

**Figure 4:**
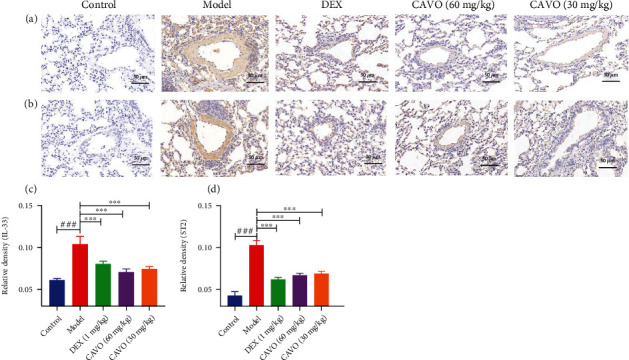
CAVO inhibits the expressions of IL-33 and ST2 in the lung tissue of asthmatic mice. The expressions of IL-33 (a, c) and ST2 (b, d) in the lung tissue were measured after treatment with CAVO or DEX. Data are expressed as mean ± SEM. (*n* = 3 mice per group, ^###^*P* < 0.001 vs. control group; ^∗∗∗^*P* < 0.001 vs. model group).

IL-2, IL-7, IL-25, IL-33, and TSLP can activate ILC2s, producing several Th2-related cytokines, such as IL-5 and IL-13. Herein, IL-2, IL-7, TSLP, IL-25, and IL-33 levels in BALF were detected using ELISA to further verify the effects of CAVO on the formation of IL-2, IL-7, IL-25, IL-33, and TSLP (Figure 3). The IL-33 and ST2 expressions were assessed via immunohistochemistry (Figure 4). The levels of IL-2, IL-7, TSLP, IL-25, and IL-33 in the BALF were significantly higher in the model group than those in the normal group. Furthermore, the expressions of IL-33 and ST2 were higher in the model group than that in the control group. Moreover, high CAVO dose significantly inhibited the production of IL-2, IL-7, TSLP, IL-25, and IL-33 in BALF of asthmatic mice (*P* < 0.001). The results of IL-33 and ST2 in immunohistochemistry were consistent with that in ELISA. These results show that CAVO can inhibit ILC2s activation by reducing the sedimentation of IL-2, IL-7, TSLP, IL-25, IL-33, and ST2.

### 3.5. CAVO Reduces Downstream Factors Related to ILC2s in the BALF of Asthmatic Mice

**Figure 5:**
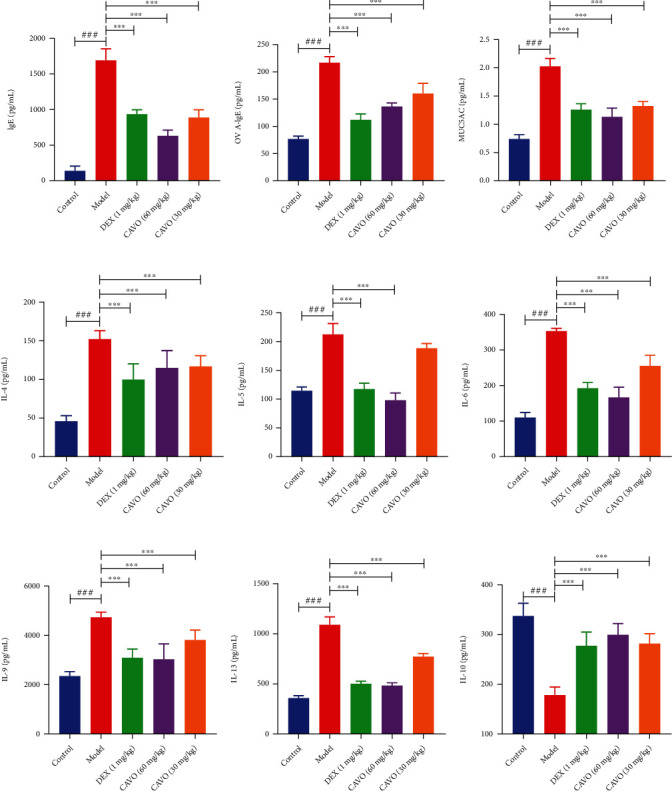
CAVO decreases the downstream factors related to ILC2s in the BALF of asthmatic mice. The levels of cytokines IgE (a), OVA-IgE (b), MUC5AC (c), IL-4 (d), IL-5 (e), IL-6 (f), IL-9 (g), IL-13 (h), and IL-10 (i) in mice serum or BALF were measured after treatment with CAVO or DEX. Data are expressed as mean ± SEM. (*n* = 6 mice per group, ^###^*P* < 0.001 vs. control group; ^∗∗∗^*P* < 0.001 vs. model group).

The production of IL-5, IL-6, and IL-13 induces the reaction of Th2 after ILC2s activation and produces inflammatory cytokines (IL-4, IL-5, IL-9, and IL-13), thus activating B cells and eosinophils. These effects can aggravate asthma progression. Herein, the expressions of IgE, OVA-IgE, MUC5AC, IL-4, IL-5, IL-6, IL-9, IL-13, and IL-10 in BALF of OVA-sensitized asthmatic mice were examined via ELISA to investigate the effects of CAVO on the production of inflammatory cytokines related to ILC2s (Figure 5). Compared with the control group, the level of IL-10 and the production of other inflammatory cytokines were significantly decreased and increased in BALF of the model group, respectively (Figure 4). However, CAVO treatment increased IL-10 levels and significantly decreased the levels of other inflammatory cytokines (*P* < 0.001). These results show that CAVO can inhibit AHR and airway inflammation by reducing the downstream factors related to ILC2s in the BALF of asthmatic mice.

### 3.6. CAVO Regulates mRNA Expression in Lung Tissue of Asthmatic Mice

**Figure 6:**
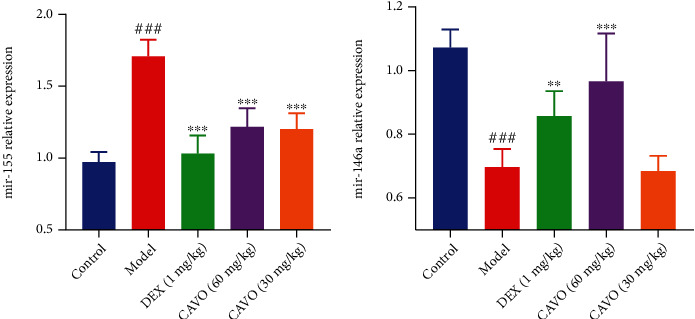
CAVO regulates the expression of mRNA in lung tissue of asthmatic mice. The expressions of miR-155 (a) and miR146a (b) in mice lung tissue were measured after treatment with CAVO or DEX. Data are expressed as mean ± SEM. (*n* = 6 mice per group, ^###^*P* < 0.001 vs. control group; ^∗∗∗^*P* < 0.001 and ^∗∗^*P* < 0.01 vs. model group).

The proliferation of ILC2s and its function of cytokine secretion are associated with miR-155 in allergen-induced inflammation. In this study, miR-155 and miR-146a expressions in lung tissue of asthmatic mice were measured via Real-Time RT-qPCR (Figure 6). Compared with the control group, miR-155 expression increased, while miR-146a expression decreased. However, after CAVO treatment miR-155 expression decreased and miR-146a expression increased compared with the model group.

### 3.7. CAVO Inhibits ILC2s in Lung Tissue of Asthmatic Mice

**Figure 7:**
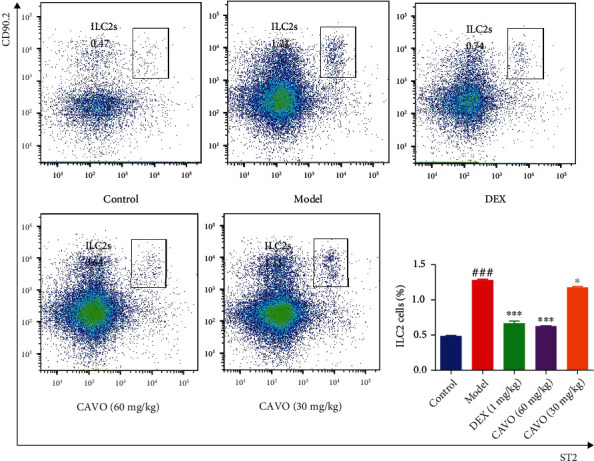
CAVO inhibits ILC2s frequency in lung tissue of asthmatic mice. ILC2s were isolated from the lung tissue of mice, and the percentages of ILC2s cells were determined using a flow cytometer. Data of column graphs were presented as means ± SEM. (*n* = 4 mice per group, ^###^*P* < 0.001 vs. control group; ^∗∗∗^*P* < 0.001 and ^∗^*P* < 0.05 vs. model group).

ILC2s can inhibit the proliferation of Th cells and control asthma. Herein, cells from the lung of asthmatic mice were analyzed via flow cytometry after 14 days of CAVO treatment to investigate the effects of CAVO on ILC2s. ILC2s were positive for CD45, CD90.2, and ST2 and negative for lineage markers (NK1.1, CD3*ε*, Ly-6G and Ly-6C (Gr-1), CD5, CD8*α*, CD11c, CD19, F4/80, FceRI*α*, and TER119). The proportion of ILC2s significantly upregulated (*P* < 0.001) in the MOD group (Figure 7). Moreover, DEX and CAVO significantly decreased the proportion of ILC2s (*P* < 0.001) compared with the model group. Interestingly, high dose of CAVO was deduced more significantly than other groups. These findings suggest that CAVO ameliorates asthmatic AHR probably by inhibiting ILC2s.

## 4. Discussion

Asthma is a recurrent chronic inflammatory disease and is significantly threatening human life and health due to the high morbidity and mortality [24]. Asthma has a complex cause, a long duration, and a high recurrence rate [25]. Asthma affects people of all ages, and its incidence is rapidly rising in developing and developed countries. Many drugs, including conventionally inhaled corticosteroids, *β*2-adrenergic drugs, methylxanthines, and IgE blockers are used to treat asthma. However, corticosteroid inhalation is considered the most effective treatment, and this is widely used [26, 27]. However, these drugs have side effects when used for long-term [28]. TCM and its ingredients have unique characteristics and advantages, and their curative effect has been verified clinically. Besides, TCM has low side effects in allergic asthma patients.

CAVO has been widely used to treat diseases of the respiratory systems in children. CAVO can protect the upper respiratory tract in the smoking mouse model. CAVO can also improve the congestive position of lung tissue and promote cell immune adjustment [23]. However, the effect and mechanism of CAVO in asthmatic animals are unknown.

In this study, OVA-induced asthmatic mice were used to evaluate the effect and mechanism of CAVO on asthmatic airway inflammation. OVA inhalation can cause AHR, mucus hypersecretion, morphological changes in lung tissues, airway goblet cell hyperplasia, and inflammatory cell infiltration in the bronchiolar walls [28]. Herein, CAVO relieved the airway resistance of asthmatic mice and induced mucus hypersecretion. CAVO also improved the morphological changes in lung tissues and airway goblet cell hyperplasia. Furthermore, CAVO alleviated inflammatory cell infiltration in the bronchiolar walls.

Asthma is associated with Th2 cells, which produce high contents of IgE antibodies in the peripheral blood. Herein, clear Th2 responses were observed in the OVA-induced asthmatic mice. Type 2 immune responses increase and cluster eosinophils in the inflammation location, which is one of the characteristics of type 2 immunopathology [29, 30]. In this study, DEX was used as a positive control drug. CAVO and DEX inhibited mucin levels and related inflammatory factors in mice BALF. CAVO and DEX significantly declined the levels of IgE, OVA-IgE, and MUC5AC.

Besides Th2 cells, ILC2s may also promote asthma development [31]. The surface of ILC2s expressed IL-25, IL-33, and TSLP receptors. Airway epithelial cells secreted IL-25, IL-33, TSLP, and other cytokines after exposure to allergens. These cytokines combined with the receptors on the ILC2s surface can rapidly activate ILC2s. IL-33, belonging to the IL-1 family, can promote bronchial remodeling and lung fibrosis by targeting various key effector cells in the adaptive immune system and innate immune system, thus further accelerating asthma development [32]. IL-33 can promote the activation and proliferation of ILC2s in vivo and in vitro via ST2 receptor signaling [33]. TSLP, a major ILC2s activator, can decrease the production of type 2 cytokines. Meanwhile, the release and production of TSLP may result in steroid resistance in moderate to severe asthma [34]. IL-25, an activated factor of ILC2s, can amplify the response of ILC2s activated by IL-33 plus TSLP/IL-2/IL-7 [35]. In this study, CAVO decreased IL-2, IL-7, IL-25, IL-33, ST2, and TSLP levels, further inhibiting ILC2s activation.

Activated ILC2s can secrete several Th2-related cytokines, such as IL-5, IL-9, and IL-13. Animal models of allergic airway inflammation showed that IL-5 participates in pulmonary eosinophilia and airway responsiveness. IL-5 is essential for the development, maturation, and survival of eosinophils and is recruited into the lungs [36]. IL-9 participates in the regulation of autoimmune and allergic reactions. IL-9 can promote the maturation of eosinophil precursors mediated by IL-5 and increase eosinophil numbers [37]. IL-9 can also regulate the maturation and aggregation of some chemokines. For example, eotaxin participates in asthma pathogenesis by promoting the differentiation, maturation, and accumulation of acidophil precursors in the lungs [38]. IL-13 enhances allergic responses because of its various functional properties. IL-13 also mediates airway hyperreactivity. IL-13 promotes macrophage activation, increases the permeability of mucus production by airway epithelial cells, and affects collagen deposition and AHR. ILC2s are a major innate source of IL-13 and are indirectly activated by allergens infiltrating the lung [39]. Meanwhile, IL-9 enhances the production of IL-5 and IL-13 in ILC2s, while activated ILC2s can secrete IgE and IgG. Herein, CAVO administration decreased IL-5, IL-6, IL-9, and IL-13 levels in BALF of OVA-sensitized asthmatic mice, indicating that CAVO inhibits inflammation in lung tissue by regulating ILC2s. IL-10 expression may have a great significance for asthma and is low in asthma [40]. Interestingly, CAVO significantly increased IL-10 levels to inhibit inflammation in lung tissue of OVA-induced mice.

LC2s are determined in humans and mice. It can be isolated from lung, mesentery, and small intestines in mice [41, 42]. ILC2s have important roles in systemic as well as local tissue immunity and are involved in early immune responsiveness and can migrate between tissues [11]. ILC2s were positive for CD45, CD90, CD127, KLRG1, and ST2 [31]. In this study, CD90+CD45+ST2+ILC2 cells were detected. OVA markedly increased the number of ILC2s, while CAVO significantly reduced the number of ILC2s, especially in the high CAVO dose group. Besides acute inflammation, miR-155 regulates a prolonged chronic inflammatory response through ILC2s. miR-155 can affect IL-33 receptor (ST2) expression, IL-33 responsiveness, and IL-13 production by directly regulating the ILC2s subset. In this study, IL-33 increased the mRNA expression of miR-155, while CAVO significantly downregulated the mRNA expression of miR-155. miR-146a is an intrinsic regulatory mechanism for ILC2s. It also affects the ability of ILC2s to secrete IL-13 and IL-5, especially the IL-33/ST2 signal pathway. Herein, CAVO downregulated miR-155, but upregulated miR-146a, suggesting that CAVO regulates ILC2s by increasing miR-155 expression and inhibiting miR-146a expression.

CAVO is an aromatic herbal medicine that has been widely used to treat asthma for many years. It is beneficial to humans and can improve effectively airway inflammation. However, the therapeutic effect and mechanism of CAVO on asthma are unknown. In this study, CAVO attenuated OVA-induced asthma in mice, inhibited the upstream inflammatory cytokines, and suppressed ILC2s activation, thus inhibiting the downstream inflammatory cytokines. Meanwhile, CAVO also inhibited the microRNA-155 expression and increased microRNA-146a expression. These results suggest that CAVO can be used to treat asthma.

**Figure 8:**
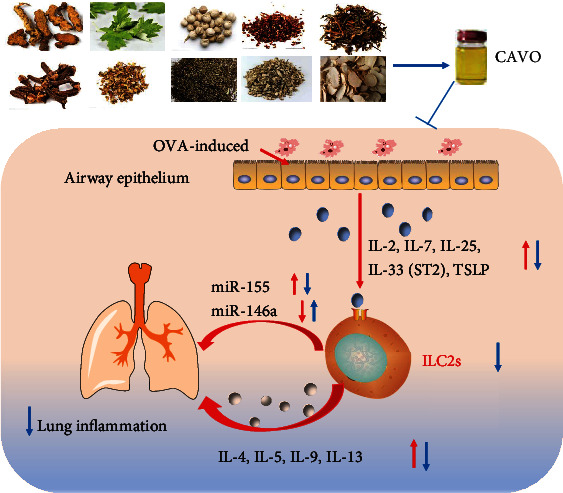
Mechanism of CAVO inhibiting ILC2s activation in OVA-induced asthma mice. CAVO improves the AHR and lung inflammation by inhibiting ILC2s. CAVO also regulates the expressions of miR-146a and miR-155. Up arrows indicate increases, and down arrows indicate decreases.

In conclusion, CAVO can significantly reduce AHR, airway inflammation, airway goblet cell hyperplasia, and mucus production. CAVO can also reduce the sedimentation of IL-2, IL-7, IL-25, IL-33, ST2, and TSLP, thus activating ILC2s. CAVO can also reduce the production of IL-4, IL-5, IL-6, IL-9, and IL-13, thus inducing the reaction of Th2. CAVO alleviates IgE, OVA-IgE, and MUC5AC, while increasing IL-10, thus decreasing airway inflammation and mucus production. CAVO deduces inflammatory effect by inhibiting the activation of the ILC2s signaling pathway in OVA-induced asthmatic mice. These findings provide a theoretical basis for the clinical application of CAVO and elucidate the mechanism of CAVO in OVA-induced asthmatic mice (Figure 8).

## Data Availability

The data presented in this study are available upon request from the corresponding author.
